# Fabrication of a Flexible Micro Temperature Sensor for Micro Reformer Applications

**DOI:** 10.3390/s110403706

**Published:** 2011-03-25

**Authors:** Chi-Yuan Lee, Chien-Hen Lin, Yi-Man Lo

**Affiliations:** Department of Mechanical Engineering, Yuan Ze Fuel Cell Center, Yuan Ze University, Taoyuan, Taiwan; E-Mails: s911415@mail.yzu.edu.tw (C.-H.L.); s995048@mail.yzu.edu.tw (Y.-M.L.)

**Keywords:** MEMS, flexible micro temperature sensor, micro reformer

## Abstract

Micro reformers still face obstacles in minimizing their size, decreasing the concentration of CO, conversion efficiency and the feasibility of integrated fabrication with fuel cells. By using a micro temperature sensor fabricated on a stainless steel-based micro reformer, this work attempts to measure the inner temperature and increase the conversion efficiency. Micro temperature sensors on a stainless steel substrate are fabricated using micro-electro-mechanical systems (MEMS) and then placed separately inside the micro reformer. Micro temperature sensors are characterized by their higher accuracy and sensitivity than those of a conventional thermocouple. To the best of our knowledge, micro temperature sensors have not been embedded before in micro reformers and commercial products, therefore, this work presents a novel approach to integrating micro temperature sensors in a stainless steel-based micro reformer in order to evaluate inner local temperature distributions and enhance reformer performance.

## 1. Introduction

Proton exchange membrane fuel cells (PEMFCs) use hydrogen and oxygen as fuel. In this process, oxygen can be produced from the atmosphere, and hydrogen can be produced from fossil fuels. The main processes involved are steam reforming, partial oxidation and coal gasification. Hydrogen can also be produced from non-fossil fuels, in which the main processes are water splitting, photoelectrochemical water splitting [1,2] and a few of other methods. Reformers are extensively used in fuel cells due to ease in obtaining fossil fuels and the large amount of hydrogen available after reforming.

Methanol is a more attractive liquid fuel for use in a proton exchange membrane fuel cell due to its high degree of safety in terms of storage and portability, high carbon/hydrogen ratio, high power density and low temperature reforming capability (250 ∼ 300 °C) [3]. However, depending on the available technology, persistent problems on the application side include the starting time, power density, and difficulty in producing hydrogen efficiently and continuously.

A flexible electrical element differs from a traditional one in that the latter uses silicon and glass as the base material, and is more fragile and prohibitively expensive. Conversely, a flexible micro electric element fabricated on thin metal foil or a high polymer is inexpensive, lightweight, shockproof and shatterproof. Due to its high mobility, a flexible electrical element can be used in different electrical product shapes and has a wide range of applications.

By using a resistance type micro temperature sensor, this work monitors the inner temperature of a micro reformer and uses physical vapor deposition to deposit Au for use as a sensitive metal. Additionally, by using a thin metal foil as the base material of a micro sensor, this work attempts to decrease the interference of a methanol reformer and diagnose the inner environment immediately. Liu and Yang used polyimide as the base material [4,5], while Chuang fabricated a micro temperature sensor and micro heater on a PDMS film [6]. This work fabricated the micro temperature sensor on stainless steel foil, which has been used successfully by the authors in previous works [7]. The advantages of stainless steel are: (1) it has better chemical resistance than other metals (non-noble metals); (2) high mechanical strength, it can be placed and operated in a high temperature and high pressure environment; (3) good heat conductivity and electric conductivity. The properties of stainless steel are listed in Table 1.

Garcia-Alonso [8] fabricated a thin film high pressure sensor on a stainless steel substrate with a SiO_2_ insulator interlayer; the process included a precipitation heat treatment, polishing and cleaning processes, and a chemical surface preparation.

Chang [9] integrated a capacitive pressure sensor on a stainless steel substrate, whereby a stainless steel sheet (12.7 μm) with a surface roughness of 100 Å and a titanium film (25.4 μm) with a surface roughness of 60 Å are laminated on the stainless steel substrate using a hot press.

Jiang [10–13] fabricated an array temperature sensor on a flow plate to measure the temperature distribution. The sensor in this example was made of silicon, is much thicker than stainless steel, and it is not flexible, thus, in our work we fabricated the sensor with stainless steel.

Shih fabricated a platinum thin film micro temperature sensor on a chip and then combined it with complementary metal-oxide semiconductor (CMOS). The measurement ranged from 25 °C to 65 °C, resistance ranged from 11.9 ∼ 12.7 KΩ, and the sensitivity was 1.68 KΩ/°C [14]. In our work, we have obtained a better and wider temperature measurement range from 30 °C to 280 °C.

Xiao [15] developed an economic and flexible micro temperature sensor by first using low pressure chemical vapor deposition to deposit phosphosilicate glass to be the sacrificial layer. A layer of polyimide was then coated, followed by use of MEMS technology to layer platinum on the polyimide, making the sensor flexible. Finally, the sacrificial layer was removed, by which the position of the sensor was not limited.

Using polyamide foil as the basic material, Bielska [16] fabricated a gold and copper electrode on it via MEMS technology. The temperature ranged from 30 °C–42 °C, and the resistance change according to the temperature was used to study its linear performance.

Although micro temperature sensors have been used in numerous areas, for example, fuel cells, lithium batteries, *etc.*, they have not been embedded yet in micro reformers and commercial products. This work describes the fabrication of micro temperature sensors on stainless steel foil using MEMS technology. The proposed fabrication approach is characterized by its compact size, flexible yet precise measurement positions, mass production capability, usability in high-temperature (>300 °C) environments, as well as high corrosion resistance and high compression resistance, capable of withstanding the operating environment of a micro reformer. Because the conversion efficiency increases with the temperature, thus, by monitoring the inner temperature, we can change the water/methanol ratio and fuel supply rate immediately, and increase or maintain the conversion efficiency as desired.

## 2. Methodology

In this work, a resistance temperature detector (RTD) was used as the temperature sensor. As a metal conductor with a positive temperature coefficient (PTC), the resistance of an RTD increases with an increasing environmental temperature. A situation in which the temperature variation of an RTD is linear suggests that the relationship between measured resistance and temperature change can be expressed as follows:

(1) $$R_{t}=R_{r}(1+\alpha_{T}\Delta T)$$

(2) $$\Delta T=t-t_{r}$$

where *R_t_* and *R_r_* denote the resistances of an RTD at *t* °C and *r* °C, respectively; α*_T_* denotes the positive temperature coefficient of RTD; *ΔT* represents the variation in a temperature relative to the reference temperature; and *t* and *t_r_* refer to the RTD temperatures at *t* °C and *r* °C, respectively. Equation (2) can thus be rewritten as:

(3) $$\alpha_{T}=\frac{R_{t}-R_{r}}{R_{r}(\Delta T)}$$

where α*_T_* denotes the sensitivity of a temperature sensor (1/°C) [17]. This micro temperature sensor is used hereinafter in the micro reformer.

## 3. Fabrication of Flexible Micro Temperature Sensors

**Figure 1.**
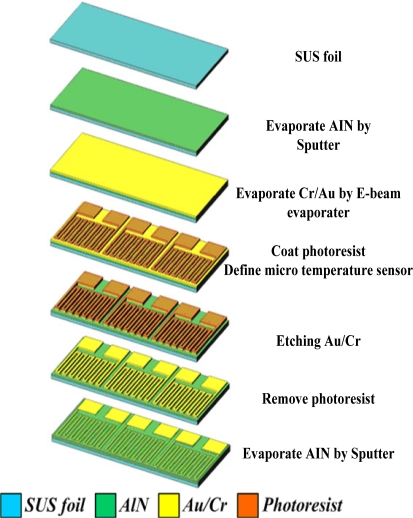
Flowchart of the micro temperature sensor assembly.

**Figure 2.**
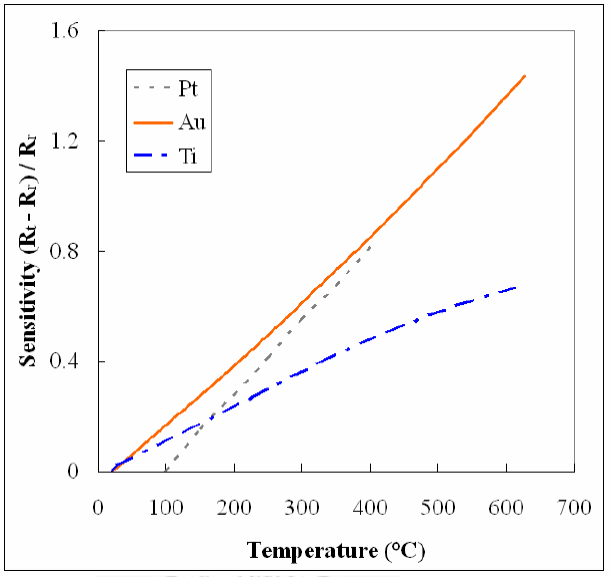
The linearity comparison of sensing materials [19,20].

Figure 1 illustrates diagrammatically the flexible micro temperature sensor fabrication process. Because of its low cost, stainless steel was chosen as the substrate. The micro temperature sensor is composed of gold. A layer of aluminum nitride (AlN) must be sputtered as an isolating insulation layer, regardless of whether wet etching or lift-off is used. AlN was adopted as an insulation layer, since it has excellent insulating and high thermally conductive properties [18]. Consequently, in this work, 10,000 Å of AlN is sputtered. Since AlN is readily etched by MP2500 developer, the pattern is defined using an etching method. Figure 2 shows the linearity of gold compared with platinum; although platinum has better linearity than gold, gold still shows a good linearity at the reformer operating temperature (below 300 °C); Table 2 shows the resistivity of gold compared with other metals, and we can see that gold has the second highest resistivity [19]. Although platinum seems better than gold, it costs much more and one has to use lift-off, which is harder to control accurately, to define the pattern. Thus, gold was adopted as the sensing material.

**Figure 3.**
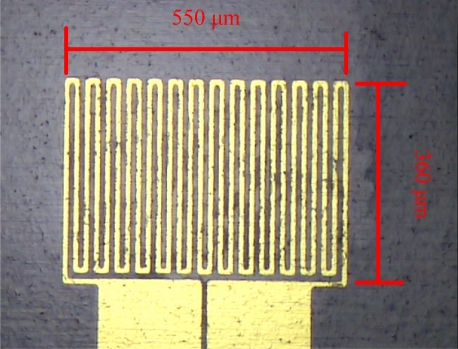
Optical microscopy image of a micro temperature sensor.

Initially, chromium (Cr, 400 Å) was evaporated as a sticking layer by an E-beam evaporator, followed by evaporation of 2,000 Å of gold. Next, the pattern of a micro temperature sensor is defined by photolithography, followed by etching of chromium and gold. Finally, the photoresist is removed with acetone. Figure 3 displays the micro temperature sensor. The details of the fabrication parameters are shown in Table 3.

## 4. Results and Discussion

The inner resistance of a temperature sensor can be obtained based on the equation relating temperature and resistance, followed by calculation of the temperature inside the reformer by interpolation.

**Figure 4.**
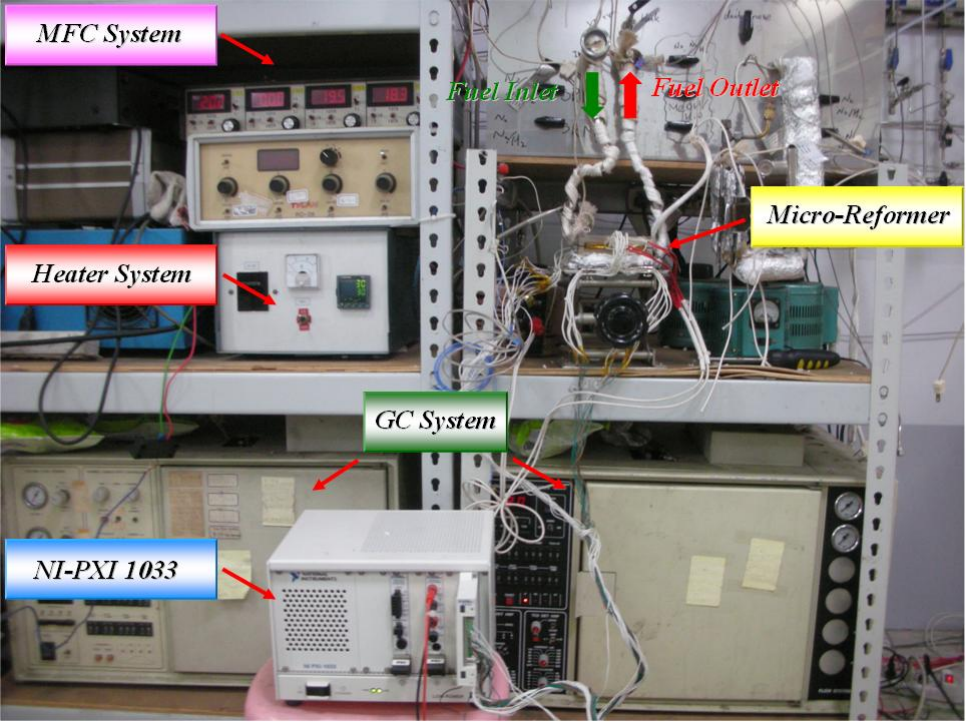
Calibration system.

**Figure 5.**
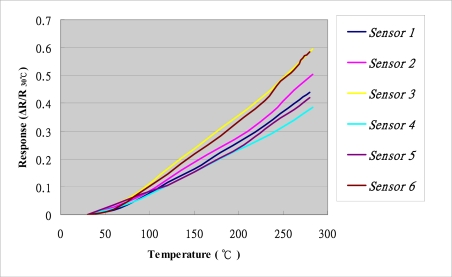
Calibration curves of micro temperature sensors.

The calibration system is shown in Figure 4. The sensor is first fabricated at the flow plate, and then is put into the drying oven chamber to control the temperature. A NI PXI 1033 unit is used to measure the resistance of the sensor, and then the sensor can be calibrated. The temperature is calibrated from 30 °C to 280 °C. Figure 5 shows the calibration curve, indicating that the temperature and resistance are linearly dependent on each other.

**Figure 6.**
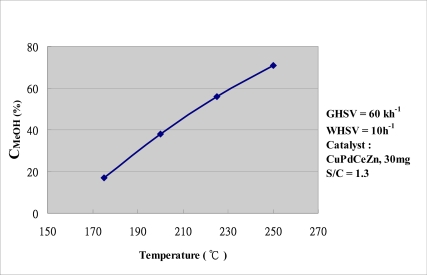
Relative curve of conversion and temperature under the SRM reaction.

Next, steam reforming of methanol (SRM) was performed with 30 mg of a catalyst, followed by infusion of a mixture of methanol and water (S/C = 1.3). Following reduction of the catalyst, the temperature is increased from 175 °C to 250 °C in 25 °C intervals, during which the relationship between reaction temperature and methanol conversion is observed. When heating to 250 °C, the methanol conversion is close to 71%. Figure 6 shows the measurement results. Because the conversion efficiency increases with the temperature, thus, by monitoring the inner temperature, we can change the water/methanol ratio and fuel supply rate immediately, and then increase or maintain the conversion efficiency.

**Figure 7.**
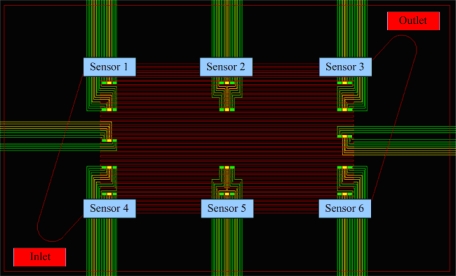
Distribution of micro temperature sensors during calibration.

**Figure 8.**
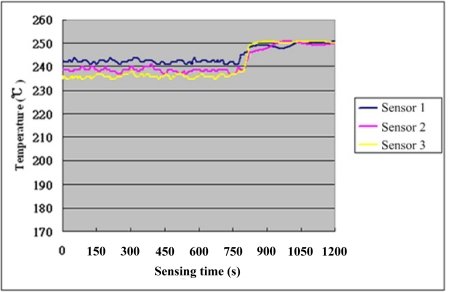
Temperature distribution of micro sensors 1 ∼ 3 insider the reformer.

**Figure 9.**
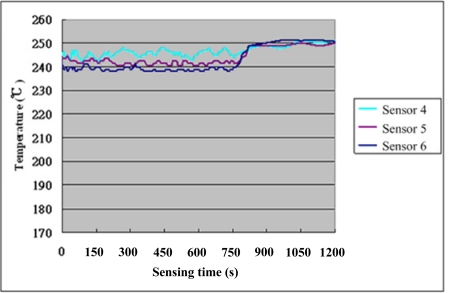
Temperature distribution of micro sensors 4 ∼ 6 insider the reformer.

After steady operation of the micro reformer at 250 °C, the NI PXI-1033 system is used to capture the signal of micro temperature sensor every 1.5 seconds, with the fuel supply stopped after 750 seconds to observe the variation in temperature. Figure 7 shows the assigned numbers and location of micro temperature sensors, which has two sensors at each part of the reformer (upstream, midstream and downstream). Consequently, the curve between temperature and time can be obtained, as shown in Figures 8 and 9. Table 4 shows the sensitivity, linearity and initial resistance of the micro temperature sensors.

## 5. Conclusions

This work describes the fabrication, calibration and measurement of a micro temperature sensor. After the catalyst is reduced, the temperature is heated in intervals of 25 °C from 175 °C to 250 °C, during which the relationship between reaction temperature and methanol conversion is observed. This work first presents a novel approach to integrating micro temperature sensors in a stainless steel-based micro reformer in order to evaluate inner local temperature distributions. The proposed fabrication approach is characterized by its compact size, flexible yet precise measurement positions, mass production capability, use in high-temperature (>300 °C) environments, as well as high corrosion resistance and high compression resistance, capable of withstanding the operating environment of a micro reformer.

## Figures and Tables

**Table 1.** The properties of stainless steel.

Specific gravity	8.03
Coefficient of linear expansion (×10^−6^/°C)	18.7/0 ∼ 650 °C
Thermal conductivity (W/m°C)	16.3
Specific heat (×10^3^ J/kg°C)	0.5
Hardness (HV)	≤200

**Table 2.** Compared resistivity of metals [21].

**Material**	**ρ (μΩ·m)**	**α_T_ (ppm/°C)**	**ρα_T_ (pΩ·m)**
Pt	0.102	3900 ± 0.9%	∼400
Au	0.0222	3950 ± 1.3%	∼90
Ag	0.0155	3950 ± 3.8%	∼60
Cu	0.0157	4200 ± 2.4%	∼70

**Table 3.** Fabrication parameters of micro temperature sensors.

**Step**	**Recipe**
1	**Steel foil:** stainless steel foil substrate (SS-304 40 μm thick) H_2_SO_4_ + H_2_O_2_ (cleaning 10 mins)
2	**AlN sputtering:** Ar (6.8 sccm), N_2_ (1.7 sccm), Pressure (0.23 Pa), Substrate temperature (120 °C), Power (150 W)
3	**Cr/Au evaporating:** Substrate temperature (100 °C), Background pressure (8 × 10^−7^ Torr), Cr thickness (200 Å), Au thickness (2,000 Å)
4	**Lithography:** Spin coated photoresist (AZ-4620)→ 6,000 rpm, 30 s. Soft bake→ 110 °C, 90 s. Exposure→182 mJ/cm^2^. Development→MP 2500:DI water = 5:1, Hard bake→ 110 °C, 10 mins
5	**Au/Cr etching:** Au etching (KI + I_2_), Cr etching (Cr-7)
6	**Lithography:** Spin coated photoresist (AZ-4620)→ 6,000 rpm, 30 s. Soft bake→ 110 °C, 90 s. Exposure→ 182 mJ/cm^2^. Development→ MP2500:DI water = 5:1, Hard bake→ 110 °C, 10 mins
7	**AlN etching:** H_3_PO_4_ (70 °C)
8	**Photoresist (AZ-4620) back coating:** Spin coated photoresist→ 1,000 rpm, 10 s. Hard bake→ 110 °C, 30 mins.
9	**Steel foil etching:** Aqua regia (40 °C)
10	**Define protection layer:** Spin coated photoresist (AZ-4620)→ 6,000 rpm, 30 s. Soft bake→ 110 °C, 90 s. Exposure→ 182 mJ/cm^2^. Development→ MP2500:DI water = 5:1, Hard bake→ 110 °C, 30 mins

**Table 4.** Sensitivity of the micro temperature sensors.

**Micro sensors**	**Sensitivity**	**Linearity**	**Initial resistance**

Sensor 1	1.8270 × 10^−3^/°C	0.993826146	517 Ω
Sensor 2	1.9949 × 10^−3^/°C	0.991182651	514 Ω
Sensor 3	2.3536 × 10^−3^/°C	0.995711337	478.9 Ω
Sensor 4	1.5261 × 10^−3^/°C	0.995731697	648 Ω
Sensor 5	1.8181 × 10^−3^/°C	0.991984453	612 Ω
Sensor 6	2.4074 × 10^−3^/°C	0.992921296	478.8 Ω

## References

[1] Minggu LJ, Daud WRW, Kassim MB (2010). An overview of photocells and photoreactors for photoelectrochemical water splitting. Int. J. Hydrogen Energy.

[2] Kelly NA, Gibson TL (2008). Solar energy concentrating reactors for hydrogen production by photoelectrochemical water splitting. Int. J. Hydrogen Energy.

[3] Brunetto C, Moschetto A, Tina G (2009). PEM fuel cell testing by electrochemical impedance spectroscopy. Electric. Power Syst. Res.

[4] Liu P, Zhu R, Que R (2009). A flexible flow sensor system and its, characteristics for fluid mechanics measurements. Sensors.

[5] Yang YJ, Cheng MY, Chang WY, Tsao LC, Yang SA, Shih WP, Chang FY, Chang SH, Fan KC (2008). An integrated flexible temperature and tactile sensing array using PI-copper films. Sens. Actuat. A.

[6] Chuang HS, Wereley S (2009). Design, fabrication and characterization of a conducting PDMS for microheaters and temperature Sens. J. Micromech. Microeng.

[7] Lee CY, Weng FB, Cheng CH, Shiu HR, Jung SP, Chang WC, Chan PC, Chen WT, Lee CJ (2011). Use of flexible micro-temperature sensor to determine temperature *in situ* and to simulate a proton exchange membrane fuel cell. J. Power Sources.

[8] García-Alonso A, Huizti X, Castaño E, Gracia FJ (1993). An optimized preparation process of stainless-steel substrates and their application to thin-film high pressure sensors. Sens. Actuat. A.

[9] Chang SP, Allen MG (2004). Demonstration for integrating capacitive pressure sensors with read-out circuitry on stainless steel substrate. Sens. Actuat. A.

[10] Jiang L, Wong M, Zohar Y A micro-channel heat sink with integrated temperature sensors for phase transition study.

[11] Jiang L, Wong M, Zohar Y (1999). Phase change in microchannel heat sink with integrated temperature sensors. J. Microelectromech. Syst.

[12] Jiang L, Wong M, Zohar Y (2000). Transient temperature performance of an integrated micro-thermal system. J. Micromech. Microeng.

[13] Jiang L, Wong M, Zohar Y (2001). Forced convection boiling in a microchannel heat sink. IEEE J. Microelectromech. Syst.

[14] Shih CY, Chen Y, Li W, Xie J, He Q, Tai YC (2006). An integrated system for on-chip temperature gradient interaction chromatography. Sens. Actuat. A.

[15] Xiao S, Che L, Li X, Wang Y (2007). A cost-effective flexible MEMS technique for temperature sensing. Microelectr. J.

[16] Bielska S, Sibinski M, Lukasik A (2009). Polymer temperature sensor for textronic applications. Mater. Sci. Eng. B.

[17] Lee CY, Lee SJ, Shen CC, Chang CC, Lin CH, Yeh CT (2010). Micro-sensors with novel micro-channels in a micro-reformer. Int. J. Hydrogen Energy.

[18] Uchiyama S, Ishigami Y, Ohta M, Niigaki M, Kan H, Nakanishi Y, Yamaguchi T (1998). Growth of AlN films by magnetron sputtering. J Crystal Growth.

[19] Chung GS, Kim CH (2008). RTD characteristics for micro-thermal sensors. Microelect. J.

[20] Zhang KL, Chou SK, Ang SS (2007). Fabrication, modeling and testing of a thin film Au/Ti microheater. Int. J. Therm. Sci.

[21] Weast RC (1983). CRC Handbook of Chemistry and Physics.

